# Timestamped list-mode data from coincidence γ-ray spectrometry with HPGe detectors on air-filter samples

**DOI:** 10.1016/j.dib.2025.111832

**Published:** 2025-06-24

**Authors:** Alf Göök, Erik Andersson Sundén, Peter Andersson, Stefan Jarl-Holm, Peter Jansson, Catharina Söderström

**Affiliations:** a Swedish Defence Research Agency (FOI), Stockholm, Sweden; b Department of Physics and Astronomy, Division of Applied Nuclear Physics, Uppsala University, Uppsala, Sweden

**Keywords:** Radionuclide monitoring, Gamma coincidence spectrometry, High purity germanium detector, BGO detector, Anti-Compton shielding

## Abstract

This data set contains raw timestamped list-mode data obtained using an array of HPGe detectors for the purpose of testing coincidence spectrometry in the context of measurement on air filter samples. Data from one air-sampling station managed by the Swedish Defense Research Agency (FOI) is made available. This air-sampling station is located in Umeå, Sweden (Latitude 63.85°N, Longitude 20.34°E, 46 m above sea level). In addition to the air filter sample, data from a blank filter as well as a filter that was spiked with a known activity of radionuclides is made available in this data set. The detector setup used to collect this data set is made up of five individual HPGe detectors, with one of them surrounded by an active BGO Compton suppression shield. The data set provides a testing ground for investigating the use of multi-fold coincidence spectrometry as a tool to lower the minimum detectable activity of anthropogenic radionuclides in air filter samples.

Access to this data set allows researchers to explore and evaluate analysis methodologies for coincidence γ-ray spectrometry on real samples.

Specifications Table

**Table:** 

Subject	Physical Sciences - Radiation
Specific subject area	Coincidence detector development for detection of atmospheric radioactivity*.*
Type of data	Raw data files in ROOT format as well as text files in CSV format produced from the raw data using an example program. All files are available in the data archive.
Data collection	The data was collected using 5 HPGe detectors, where one was situated in an anti-Compton shield consisting of BGO detectors. Timestamped list-mode data was collected for four different samples. The data was acquired using a CAEN V1782, 8-channel digital MCA. The MCA was controlled using the CoMPASS software, which was also used to write the data to disc. The DPP-PHA firmware on the digital MCA was used to treat the detector signals in order to extract the information on deposited energy and time of arrival, which is stored in the list-mode data.
Data source location	The air filter sample was collected in Umeå, Sweden (Latitude 63.85°N, Longitude 20.34°E, 46 m above sea level). The data was collected at Uppsala University. Data from the measurement is stored at the Swedish National Data Service (SND).
Data accessibility	Repository name: Swedish National Data Service (SND) Data identification number: DOI: 10.57804/0tjh-3b49 Direct URL to data: https://doi.org/10.57804/0tjh-3b49
Related research article	None

## 1 Value of the Data

• This data set provides a testing ground for investigating the use of multi-fold coincidence spectrometry as a tool to lower the minimum detectable activity of anthropogenic radionuclides in air filter samples.
• Access to this data set allows researchers to explore and evaluate analysis methodologies for coincidence γ-ray spectrometry on real samples.
• The data set provides an opportunity for researchers to validate codes used to predict efficiency of coincidence detection setups.
• The data set also provides information on the influence of background on an unshielded coincidence setup.

## 2 Background

A ban of nuclear test explosions constitutes an effective measure of nuclear disarmament and non-proliferation. Radionuclide monitoring is a proven means of effective non-intrusive verification of a nuclear test ban. The majority of radionuclide monitoring is based on detection of radioactive aerosols which are sampled using air filters and examined using gamma spectrometry with High Purity Germanium (HPGe) detectors. The precise energies of the γ-rays emitted by the sample are fingerprints of the radionuclides it contains. Detection of a radionuclide is based on counting a number of characteristic γ-rays recorded in the detector. However, the presence of background radiation affect the minimum detectable activity (MDA) [1], below which one cannot expect to detect the presence of a nuclide in the sample given the geometry of the experimental setup.

Coincidence detection is a method to lower the MDA, making monitoring more sensitive [2]. This technique could be valuable in gamma spectrometry used for radioactive particle detection, for any nuclides that emit multiple γ-rays in cascade. The technique requires the use of multiple detecting elements, e.g. in an array of detector systems or in a segmented detector, and only counting events where at least two characteristic γ-rays are simultaneously registered inside a coincidence time window. The probability that γ-rays from background sources combine to match multiple energy coincidence criteria is small. The present data set was compiled to test coincidence γ -ray spectrometry on realistic air samples. The data is suitable for developing or testing spectral analysis methods. Further use of atmospheric data and analysis is needed in combination with such spectral data and analysis methods to determine source location or activity.

## 3 Data Description

The data set comes from experimental measurements of real air-filter samples with five HPGe γ-ray detectors. Timestamped list-mode data has been collected for four different samples and is available in the data repository [8].

The data set contains measurements of four categories; see the Experimental Design, Materials and Methods section for a detailed description of the individual samples. The root directory of the repository contains one sub-directory for each of these categories. Each of these directories contains sub-directories for each individual run. For some of the samples the data collection was started and stopped several times, due to external circumstances, such as filling the HPGe detector with liquid nitrogen. Each period between a start and a stop constitutes a run. Each run directory contains files named run.info and settings.xml. The file run.info is an ASCII file that contains general run information, e.g. start timestamp, stop timestamp and counting time, while the file settings.xml contains settings of the data acquisition hardware during the run. The content of the settings.xml file is the same for all runs, since the settings did not change between them. For detailed information about the settings files we refer to the CoMPASS user manual [3]. Besides these files, each run directory contains two subdirectories with the following content:

• CSV: Contains a single file in CSV format. The content of this file is the raw list mode data translated to CSV format from the binary ROOT format.
• RAW: Contains one or several files with .root extension. This is the raw list mode data in the original binary ROOT-tree format written by the data acquisition software. When the directory contains more than a single .root file these are sequenced files from the same run. The first file in the sequence consists of a base name and ends with “.root” (e.g. base_name.root). Consecutive filenames in the sequence ends the base name with “_N.root”, where N specifies the number in the sequence (e.g. base_name_1.root).

The original raw data is stored in binary ROOT-tree format that can be read using the ROOT software [6]. For convenience, we provide an example data program to read the ROOT formatted files, written in C++ along with the data. The example source code for the program is placed in a folder named “root_data_read_example” in the root of the data archive. The compiled executable will, given a list of names of ROOT files, loop over the tree and print the content to screen. The format printed to screen is the same as the CSV-formatted data that can be found in the subdirectory of each run.

In each ROOT file, the data is stored in a tree, represented by the TTree class, with the name “Data_R”.

Each tree consist of a list of six independent columns, called branches, represented by the TBranch class and are described in Table 1. The tree format is a list-mode data format, where each line corresponds to a recorded pulse in one of the detectors in the setup.

**Table 1:** Description of the branches contained in the TTree objects.

Branch name	Data type	Description
Channel	unsigned short int (2 bytes)	The channel number on the digitizer. See Table 2 for information about the correspondence between detector and channel number.
Timestamp	unsigned long long int (8 bytes)	The timestamp of the event, i.e. the time since the start of the run. The unit for the time is pico-seconds.
Energy	unsigned short int (2 bytes)	The recorded pulse-height of the event. The pulse-height is proportional to the energy deposited in the detector by a charged particle interaction within it.
Flags	unsigned int (4 bytes)	Hexadecimal number that represent additional information about the collected event.
Probe	unsigned short int (2 bytes)	Not used.

In the translated CSV format, the Probe branch has been removed, as it does not contain pertinent information. The CSV files therefore contains four columns, the first line in each CSV-file lists column names with a one-to-one correspondence to the branch names listed in Table 1. The unit for the branch Timestamp has been changed from ps to ns in the CSV-format. This conversion does not result in any loss of information, because the timestamp clock runs at 1 GHz and the last three digits of the timestamp given in picoseconds are always zeros.

## 4 Experimental Design, Materials and Methods

### 4.1 Measured Samples

The data in the subdirectory *sample* is from a measurement of a glass fiber filter from an air-sampling station of the Swedish Defense Research Agency (FOI). The air-sampling station [4] is located in Umeå, Sweden (Latitude 63.85°N, Longitude 20.34°E, 46 m above sea level). The flow rate for the air-sampling station is about 1000 m^3^/h. The sample consists of two filters, where ¾ of each filter was compressed into a cylindrical shape, 60 mm diameter and 13 mm height. The integrated airflow through the filters was (110583±1534) m^3^ and the sample’s weight was 36.6 g. The compressed sample was placed in a polystyrene canister with a conical frustum shape, with a height of 13 mm, a maximum diameter of 65 mm and a minimum diameter of 64 mm.

The data in the subdirectory *blank* is from a measurement on a glass fiber filter of the same type as that from the air-sampling station, but through which no air was filtered. The blank filter was compressed in the same way as described above. Data from the blank filter measurements can be used to estimate the radioactivity background in the facility and from the filter material.

The data in subdirectory *calibration* is from a measurement of a similar glass-fiber-filter sample as the blank filter, but which was spiked with a known activity of radionuclides as described in Appendix 1 of Ref. [7]. The known activity was deposited by hand in a point-by-point grid pattern on the filter using a pipette before it was compressed, thereby approximately homogenously distributing the activity.

### 4.2 Experimental Setup

**Fig. 1:**
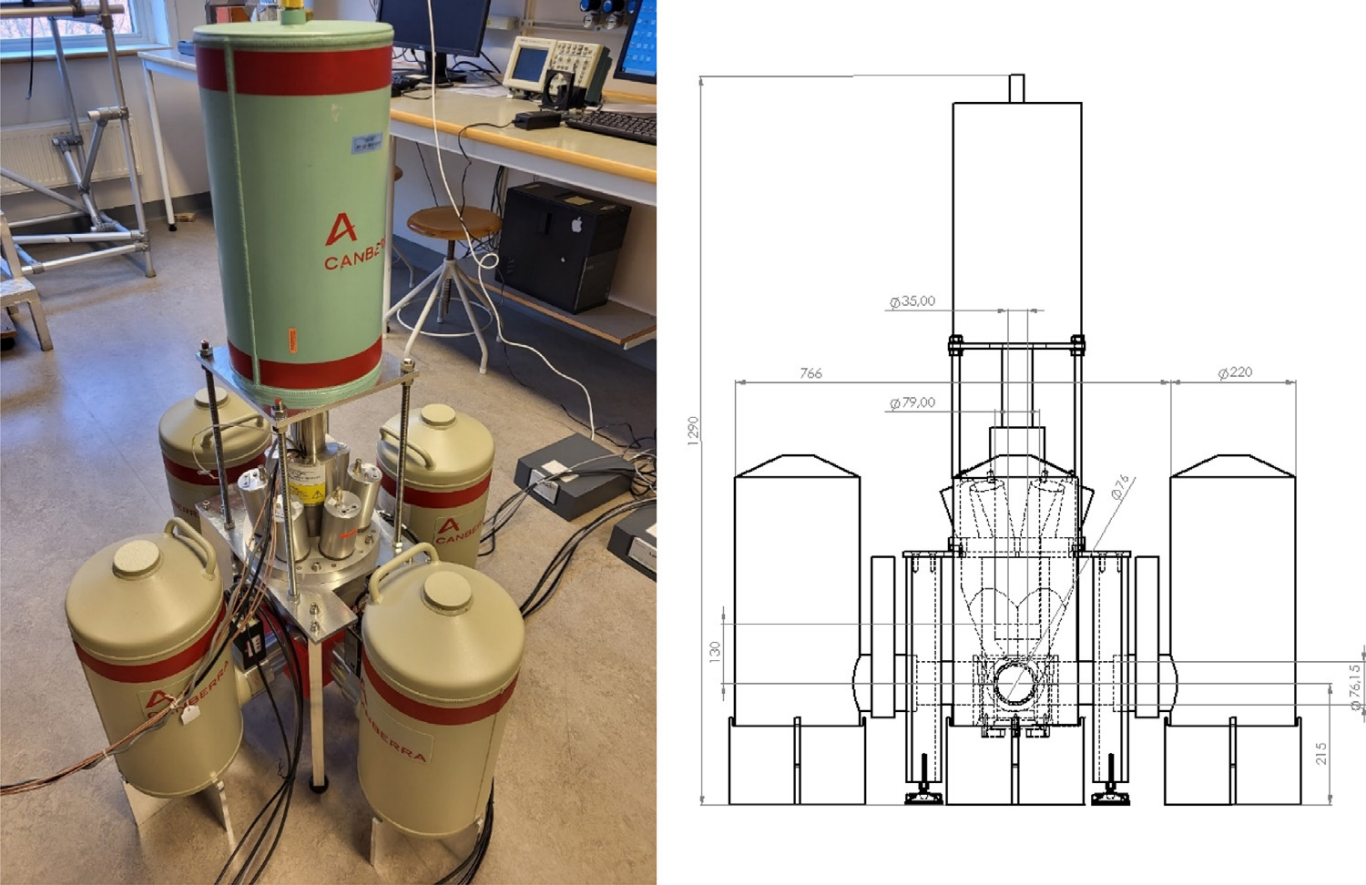
Left: Photograph of the detector setup. Right: The setup annotated with sizes and distances in mm.

**Fig. 2:**
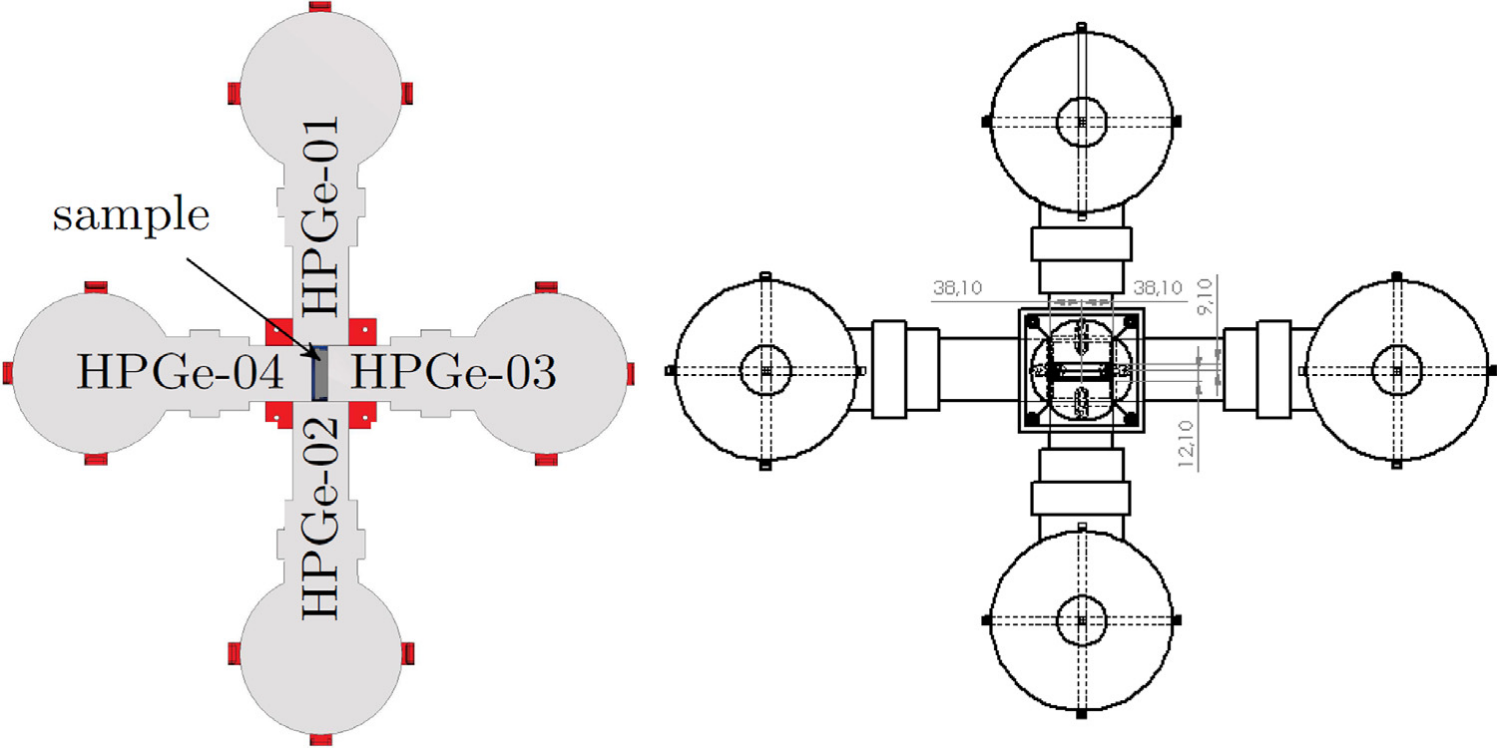
Cross-section through the center of the sample of the experimental setup, as seen from above. Left: The dark grey cylindrical shape labelled sample represents the canister containing the filter sample. The four light grey shapes represent the four HPGe-detectors that were placed in the horizontal plane. All of these detectors were placed with the front face as close as possible to the sample canister. Right: The distance from the front face of HPGe-04 to the canister lid was 3.8 mm, the distance from the front face of HPGe-03 to the canister bottom was 0.15 mm, while the distances from the front faces of HPGe-01 and HPGe-02 to the center of the canister was 38.1 mm. Individual detector parameters are summarized in Table 2. In addition to the four HPGe detectors depicted in this figure, the detector labelled HPGe-05 was located above the centre of the sample canister, with its front towards it.

A photograph of the experimental setup used to acquire the data set is displayed in Fig. 1. It consist of five high purity germanium (HPGe) γ-ray detectors and one Bismuth Germanate (BGO) anti-Compton shield. Samples were placed in the dedicated sample holder in the center of the detector setup, a drawing of the sample holder is shown in Fig. 2.

The HPGe detectors were placed asymmetrically around the samples. Four of them were placed in the horizontal plane surrounding the sample, a schematic view of these detectors placement around the sample is displayed in Fig. 2. The fifth detector was placed above the sample in a vertical orientation and was surrounded by the BGO detector. The intent is to use the BGO detector in anti-coincidence with the fifth HPGe detector to suppress the Compton continuum of the resulting γ-ray spectrum. The physical dimensions of the HPGe detectors are summarized in Table 2.

**Table 2:** Summary of the HPGe detectors used in the setup. The first column is the detectors’ name, see Fig. 2 for placement information. The second column is the channel number in the data acquisition, which is also written for each event in the list-mode data. The third and fourth columns give the dimensions of the HPGe crystal, while the fifth column is the distance from the front face of the crystal to the closest position of the detector casing.

Name	Channel	HPGe crystal dimensions [mm]		
		Diameter	Length	Distance from window
*HPGe-01*	*1*	*48*	*45*	*6*
*HPGe-02*	*2*	*45*	*33*	*6*
*HPGe-03*	*3*	*45.5*	*35*	*5*
*HPGe-04*	*4*	*45.5*	*35.5*	*5*
*HPGe-05*	*5*	*64*	*65*	*5*

### 4.3 Data Acquisition

The data was acquired using a CAEN V1782, 8-channel digital MCA. The MCA was controlled using the CoMPASS software, which was also used to write the data to disc. The DPP-PHA firmware on the digital MCA was used to treat the detector signals in order to extract the information on deposited energy and time of arrival, which is stored in the list-mode data. In the DPP-PHA firmware energy information is extracted from a detector signal using a trapezoidal filter [5]. All settings for the data acquisition is retrievable from the settings.xml files available in the data repository, cf. sect. Data Description.

**Fig. 3:**
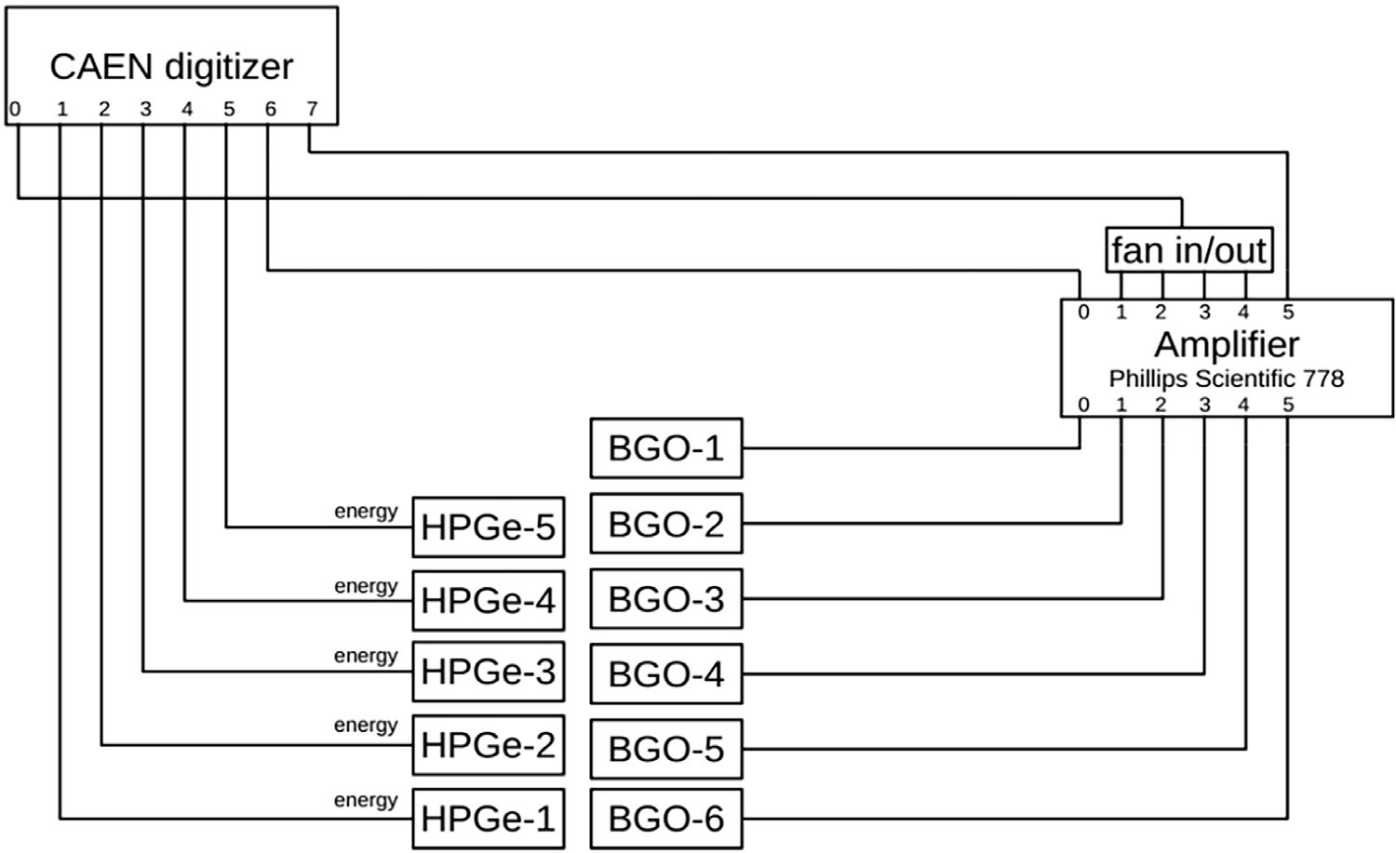
Schematic coupling scheme of the electronics used in the data acquisition.

A schematic coupling scheme for the detector electronics is displayed in Fig. 3. For all HPGe detectors in the setup, the raw pre-amplifier signal was fed directly to the digital MCA channels 1-5. The detector signal treatment for the BGO detector differs from the HPGe signal treatment. The BGO detector delivers one output signal for each individual photo-multiplier tube (PMT). Since only three channels on the MCA was available, the six output signals needed to be summed before being digitized. To this end, each PMT signal was first fed to an individual channel on a Phillips Scientific 778 preamplifier, with adjustable gain and offset. The preamplifier also reshapes the PMT signals to be better compatible with the digital signal treatment of the DPP-PHA firmware of the MCA. Four out of the six PMT signals were successfully gain-matched by utilizing a ^137^Cs γ-ray source. These four channels all showed very similar characteristics in terms of energy resolution and gain in the PMTs. Consequently, they were chosen to be summed, using a linear fan in/out. The remaining two PMT signals were digitized individually.

## Limitations

None

## Ethics Statement

The authors have read and follow the ethical requirement for publication in Data in Brief and confirms that the current work does not involve human subject, animal experiments, or any data collected from social media platforms.

## CRediT authorship contribution statement

**Alf Göök:** Conceptualization, Software, Writing – original draft, Visualization, Investigation. **Erik Andersson Sundén:** Conceptualization, Writing – review & editing, Investigation. **Peter Andersson:** Conceptualization, Project administration, Funding acquisition, Investigation. **Stefan Jarl-Holm:** Resources, Visualization. **Peter Jansson:** Conceptualization, Software, Data curation, Writing – review & editing, Project administration, Funding acquisition. **Catharina Söderström:** Resources, Writing – review & editing.

## Data Availability

Swedish National Data Service (SND)Data for: Timestamped list-mode data from coincidence γ-ray spectrometry with HPGe detectors on air-filter samples (Original data).
